# Iron Chlorin E6 enhances drought resilience in *Camellia oleifera* Abel

**DOI:** 10.3389/fpls.2025.1666016

**Published:** 2025-09-15

**Authors:** Dayu Yang, Zhigang Li, Yimin He, Xiaofan Ma, Jiancai Shen, Chengfeng Xun, Zhen Zhang, Rui Wang, Yongzhong Chen, Zhilong He

**Affiliations:** ^1^ Research Institute of Oil Tea Camellia, Hunan Academy of Forestry, Changsha, China; ^2^ Yuelushan Laboratory, Changsha, China; ^3^ National Engineering Research Center for Oil Tea Camellia, Changsha, China; ^4^ State Key Laboratory of Woody Oil Resources Utilization, Changsha, China

**Keywords:** drought stress, iron dihydroporphyrin, oil-tea camellia, plant growth regulators, ICE6

## Abstract

This study investigates the effects of Iron Chlorin E6 (ICE6) on the physiological and biochemical responses of *Camellia oleifera* plants under both normal and drought stress conditions. Varying concentrations of ICE6 (0 µg/L, 20 µg/L, 40 µg/L and 80 µg/L) were applied to *C. oleifera* plants to assess physiological parameters, including photosynthetic rate, chlorophyll content (SPAD, Soil Plant Analysis Development chlorophyll meter), antioxidant enzyme activity, proline content (Pro), soluble sugars (SS), soluble proteins (SP), abscisic acid (ABA) levels and gene expression. Under well-watered conditions, 80 µg/L ICE6 significantly enhanced net photosynthetic rate (Pn) and SPAD values by 113.73% and 11.57%, respectively, compared to the control, while promoting indole-3-acetic acid (IAA) and soluble protein accumulation. Under drought stress, ICE6-treated plants exhibited improved stress tolerance, with reduced malondialdehyde (MDA) content by 18.32% (20 µg/L), 14.14% (40 µg/L), and 30.84% (80 µg/L) compared to the control, alongside enhanced antioxidant enzyme activities, ABA signaling, and proline accumulation, mitigating oxidative damage. Among the concentrations tested, 80 µg/L ICE6 delivered the greatest overall benefit by achieving the highest net photosynthetic rate (113.73%) and SPAD value (11.57%) under well-watered conditions, and the lowest MDA accumulation (30.84%) under drought, thus identifying it as the optimal dose in this study. This study provides a theoretical foundation for the use of ICE6 as a multifunctional plant growth regulator in *C. oleifera* and offers innovative strategies to enhance drought resistance in this economically significant crop.

## 1 Introduction

*Camellia oleifera* Abel., a small evergreen tree belonging to the Theaceae family and *Camellia* genus, is one of the four major woody oil crops worldwide, alongside olive, oil palm, and coconut trees (Gao et al., 2024). The primary product of *C. oleifera*, camellia oil, is renowned for its rich nutritional profile including bioactive compounds such as squalene, phytosterol, polyphenols and fat-soluble vitamins (Vitamins A, B, E), often earning it the title of “olive oil of the East” (Shi et al., 2020). However, due to the ongoing effects of global warming, drought stress has emerged as one of the most significant abiotic challenges faced by *C. oleifera*. Drought not only affects the growth and development of *C. oleifera* but also represents a major limiting factor for yield production (He et al., 2020; Shen et al., 2024).

Under drought stress, plants typically experience a decline in photosynthetic efficiency due to stomatal closure, reduced chlorophyll content, and impaired electron transport in photosystems (Karami et al., 2025). Water deficit also leads to excessive accumulation of reactive oxygen species (ROS), which can damage cellular membranes, proteins, and nucleic acids (Gorni et al., 2025). To mitigate oxidative damage, plants activate their antioxidant defense systems, including enzymes such as superoxide dismutase (SOD), peroxidase (POD), and catalase (CAT) (Khan et al., 2025). Meanwhile, osmotic adjustment substances like proline (Pro) and soluble sugars accumulate to maintain cellular osmotic balance and protect macromolecules (Sun et al., 2025). Drought stress also disrupts hormone homeostasis, commonly elevating abscisic acid (ABA) levels to regulate stomatal closure and stress signaling, while often altering auxin (IAA) distribution, which can inhibit normal growth and development (Basso et al., 2025; Ndombi et al., 2025). Lipid peroxidation, reflected by increased malondialdehyde (MDA) content, is another typical marker of drought-induced oxidative stress (Krishankumar et al., 2025; Ying et al., 2025).

In recent years, numerous studies have investigated strategies to improve the growth and drought tolerance of *C. oleifera*, with a particular focus on using exogenous plant regulators to enhance drought resistance. Prior research has shown that pre-harvest ethylene treatments can promote the accumulation of linoleic acid (LA) and *α*-linolenic acid (ALA) in *C. oleifera* fruit, with a 1.5gL^−1^ treatment increasing LA and ALA content by 18.0% and 19.1%, respectively, compared with the control (Li et al., 2023). Hydrogen cyanamide (0.5%) has also been found to significantly extend the flowering period of *C. oleifera* (by approximately 12 days) (Lin et al., 2022). Additionally, exogenous calcium (Zhang et al., 2023) and abscisic acid (ABA) (Yang et al., 2024) have been shown to boost photosynthesis, enhance antioxidant enzyme activities and osmotic regulation in *C. oleifera* seedlings under drought, thus enhancing drought resistance. The application of plant growth regulators, which chemically influence the growth and development of crops, has become an essential practice in modern agriculture.

Iron Chlorin E6 (ICE6), also known as iron dihydroporphyrin, is a novel natural plant growth regulator that can delay chlorophyll degradation by inhibiting chlorophyllase, thereby increasing chlorophyll content (Chen, 2018). Previous research has demonstrated that ICE6 not only promotes plant growth but also acts as a novel plant immunity inducer, significantly enhancing resistance to waterlogging, salt, and drought stresses in plants (Cao et al., 2016; Chen et al., 2023; Li et al., 2024). Furthermore, recent studies have shown that seed soaking in ICE6 at 100 – 200mgL^−1^ for 24 – 48h can enhance rice seed vigor and increase germination rate (Xie et al., 2022). These findings highlight the broad potential applications of ICE6 as a multifunctional plant regulator.

Despite its promising capabilities, no studies have explored the application of ICE6 in *C. oleifera*. Therefore, this research aims to investigate the physiological and biochemical responses of *C. oleifera* plants to foliar application of ICE6 under both normal and drought conditions. By measuring various physiological and biochemical parameters, including photosynthesis, chlorophyll content, antioxidant enzyme activity, and gene expression, this study will systematically analyze the regulatory role of ICE6 in *C. oleifera* saplings. Ultimately, this work seeks to address the current gap in understanding the potential application of ICE6 to enhance drought resistance in *C. oleifera*, offering new insights into its broader agricultural potential.

## 2 Materials and methods

### 2.1 Materials

Uniform, healthy, three-year-old potted plants of *C. oleifera* ‘Xianglin 210’ were supplied by the National Engineering Research Center for Oil-tea Camellia (Changsha, China). The seedlings were cultivated in plastic pots (top diameter: 24 cm; height: 20 cm) filled with a homogenized substrate of lateritic red soil (collected from Changsha, Hunan; 112°58′ E, 28°12′ N) and peat soil (Pindstrup Mosebrug A/S, Ryomgaard, Denmark) at a 3:1 (v/v) ratio. At the beginning of the experiment, seedlings had an average height of 75.9 cm and a basal stem diameter of 7.9 mm. The experiment was conducted in the Center’s greenhouse (113°01′ E, 28°06′ N), where daytime and nighttime temperatures were maintained at 30°C and 25°C, respectively, with a relative humidity of 80–85%. Water-soluble iron dihydroporphyrin powder was purchased from Anqing baite Biology Engineering Co., Ltd. (Anqing, China), dissolved to prepare solutions of 20, 40, and 80 µg/L, and applied as foliar sprays. Plants were sprayed with a handheld atomizer until leaf surfaces were uniformly wetted to the point of slight runoff, ensuring consistent coverage across treatments. Treatments commenced on day 1 of the experiment and were repeated at 15-day intervals for a total of three applications. SPAD values were measured on intact leaves using a SPAD-502 Chlorophyll Meter (Konica Minolta, Osaka, Japan), which estimates relative chlorophyll content based on leaf transmittance at 650 nm and 940 nm.

### 2.2 The role of ICE6 in the physiological and biochemical responses of *C. oleifera* plants under normal conditions

**Figure 1:**
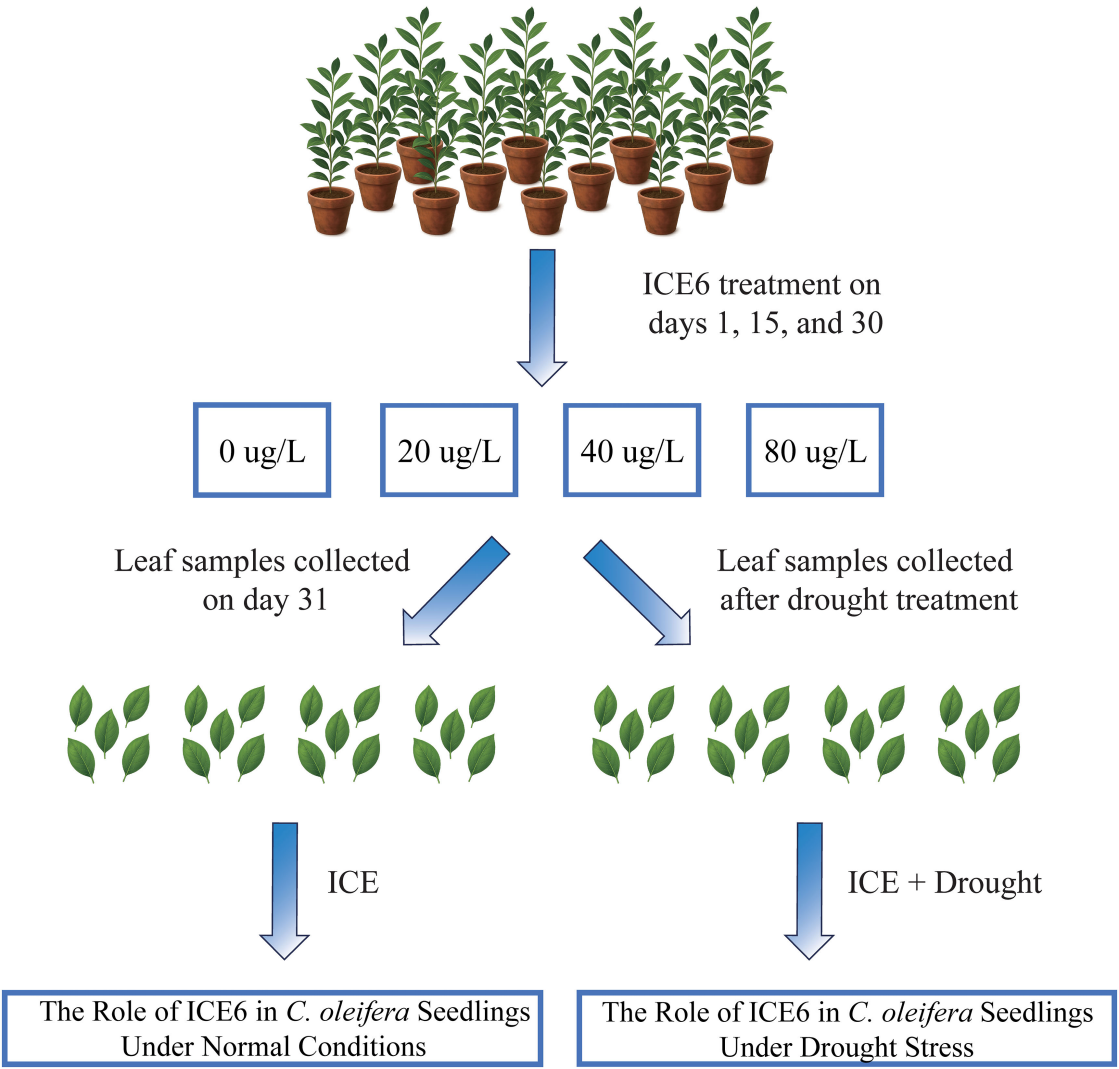
Experimental design to explore the role of ICE6 in *C. oleifera* under normal and drought stress conditions.

To investigate the physiological and biochemical responses of *C. oleifera* saplings under normal conditions following ICE6 treatment, four treatment groups with different ICE6 concentrations were established: 0 µg/L (T0, control), 20 µg/L (T1), 40 µg/L (T2), and 80 µg/L (T3). Throughout the normal condition experiment, all plants were maintained under normal growth conditions with adequate watering to avoid any water stress. ICE6 treatments were applied on days 1, 15, and 30 (**Figure 1**). Each treatment was performed with three biological replicates, and each replicate consisted of five uniformly sized plants. On day 31, photosynthetic parameters (n = 15) and SPAD values (n = 15) were measured on the fully expanded middle leaves of each plant between 9:00 and 11:00 in the morning. Subsequently, the same leaves were randomly collected, immediately frozen in liquid nitrogen, and transported back to the laboratory for further analysis. The indices measured included soluble protein (SP) content, soluble sugar (SS) content, indole-3-acetic acid (IAA) content, malondialdehyde (MDA) content, and proline (Pro) content (n = 3).

### 2.3 The role of ICE6 in the physiological and biochemical responses of *C. oleifera* plants under drought stress

Building upon the experiment described in Section 2.2, a drought stress treatment was further applied to the potted plants (**Figure 1**). The soil water content was measured using a ProCheck handheld multifunction reader/data logger. Drought stress was induced by withholding water on day 31, allowing the soil to dry naturally. Sampling was performed on the seventh day (On day 38), when the relative soil water content had decreased to approximately 30%. Once the target soil moisture level was reached, photosynthetic parameters (n = 15) and SPAD values (n = 15) were measured on the fully expanded leaves in the middle each plant between 9:00 and 11:00 in the morning. Same leaves were then collected, immediately frozen in liquid nitrogen, and transported back to the laboratory for subsequent physiological and biochemical analyses. The indices measured included superoxide dismutase (SOD) activity, peroxidase (POD) activity, catalase (CAT) activity, malondialdehyde (MDA) content, proline (Pro) content, soluble sugar (SS) content, abscisic acid (ABA) content, and soluble protein (SP) content (n = 3).

### 2.4 Measurement of photosynthetic parameters

Photosynthetic parameters, including net photosynthetic rate (Pn), intercellular CO_2_ concentration (Ci), stomatal conductance (Gs), and transpiration rate (Tr), were measured using a 6400XT Portable Photosynthesis System (LI-COR, Lincoln, NE, USA) on functional leaves of randomly selected *C. oleifera* saplings. The system was configured with an airflow rate of 500*µ*mol/s, a photosynthetically active radiation (PAR) intensity of 1000*µ*mol/m^2^/s, a CO_2_ concentration of 400 µmol/mol. Water use efficiency (WUE) was calculated as the ratio of Pn to Tr, to assess the efficiency of water utilization under specific conditions.

### 2.5 Measurement of physiological and biochemical indices

The activities of peroxidase (POD), superoxide dismutase (SOD) and catalase (CAT), together with the contents of malondialdehyde (MDA), proline (Pro), soluble sugars(SS) and soluble proteins (SP), were determined using commercial 96-well microplate assay kits (Quanzhou Ruixin Biotechnology Co., Ltd., Quanzhou, China). The following kits were used: Superoxide Dismutase (SOD) Activity Assay Kit (WST-8 method; Cat. No. G0101W), Peroxidase (POD) Activity Assay Kit (Cat. No. G0107W), Catalase (CAT) Activity Assay Kit (Cat. No. G0105W), Malondialdehyde (MDA) Content Assay Kit (Cat. No. G0109W), Proline (Pro) Content Assay Kit (Cat. No. G0111W), Soluble Sugar Content Assay Kit (Cat. No. G0501W) and Soluble Protein Content Assay Kit (Coomassie Brilliant Blue method; Cat. No. G0417W).

### 2.6 Quantification of IAA and ABA

Endogenous indole-3-acetic acid (IAA) and abscisic acid (ABA) were quantified by ultra-performance liquid chromatography–tandem mass spectrometry (UPLC–MS/MS). For each treatment, three biological replicates were prepared. Leaves from five uniformly sized plants were pooled per replicate. Each pooled sample was ground in liquid nitrogen, thoroughly mix, and accurately weigh approximately 0.3 g (to 0.0001 g) into a 15 mL centrifuge tube. Add 5 mL of extraction solution (isopropanol:water:formic acid, 80:19:1, v/v/v), homogenize for 2 min, and perform ultrasonic extraction at 4°C for 1 h. Centrifuge the extract at 10,000 rpm for 10 min to obtain the supernatant. Subsequently, add 1 mL of dichloromethane, perform low-temperature ultrasonic extraction for 30 min, and centrifuge at 10,000 rpm for 10 min to collect the supernatant. Repeat the ultrasonic extraction once more, and combine the three supernatants. Evaporate the combined supernatant under reduced pressure with nitrogen at room temperature to the aqueous phase, adjust the volume to 2 mL, vortex to mix, dilute 2-fold with methanol, and filter through a 0.22 µm membrane for analysis. All operations are conducted on wet ice and protected from light.

Chromatographic separation was performed on a Waters FTN UPLC system equipped with a BEH C_18_ column (2.1 × 50 mm, 1.7 µm). The column temperature was maintained at 40°C and the autosampler at 10°C. The mobile phase consisted of 0.1% formic acid in water (A) and acetonitrile (B), delivered at 0.3 mLmin^−1^ using the following gradient: 0.0 min, 80/20 (A/B); 1.5 min, 65/35; 3.0 min, 10/90; 4.5 min, 10/90; 4.7 min, 65/35; and 5.0 min, 80/20. The total runtime was 5 min, followed by a 5 min post time at the initial conditions (80/20) for column re-equilibration. Injection volume was 10 µL.

Mass spectrometric analysis was performed on an AB SCIEX 4000 triple quadrupole mass spectrometer equipped with an electrospray ionization (ESI) source operated in both positive and negative modes (ESI±). The MS/MS parameters were set as follows: curtain gas (CUR) 35 psi; collision gas (CAD) 7 psi; IonSpray voltage +5500 V in positive mode and –4500 V in negative mode; source temperature 450°C; ion source gas 1 (GS1) 40 psi; and ion source gas 2 (GS2) 50 psi.

The original chromatograms, calibration curves, and quantification data of IAA and ABA from UPLC–MS/MS analyses are included in the supplementary file (**Supplementary Data S1**).

### 2.7 Real-time quantitative PCR analysis

Total RNA was extracted from *C. oleifera* leaves (n = 3) using the RNAprep Pure Plant Plus Kit (TIANGEN, Beijing, China). First-strand cDNA was synthesized using the HiScript III 1st Strand cDNA Synthesis Kit (+gDNA wiper) (Vazyme, Nanjing, China), followed by five-fold dilution of the cDNA for quantitative real-time PCR (RT-qPCR) analysis. The ChamQ Universal SYBR qPCR Master Mix (Vazyme, Nanjing, China) was used for the RT-qPCR assays. Primers for the RT-qPCR were designed using Primer Premier 5 software (Premier Biosoft, Palo Alto, CA, USA), and the sequences of the primers are shown in **Table 1**. The 2{sp}−ΔΔ{it}Ct {/sp}{/it}method was used to calculate the relative expression levels of the genes. Tubulin was used as the reference gene, as it has been validated as a stable housekeeping gene in *C. oleifera* (Zhang et al., 2025). Descriptions of gene functions are provided in the supplementary file (**Supplementary Data S2**).

**Table 1:** Gene primer sequences used for the quantitative real-time PCR analysis.

Gene	Forward primer (5’-3’)	Reverse primer (5’-3’)
*Tubulin*	TGTGGAGGACGAAGAAGATGG	TCAAGACAGAGAATGGCAATACC
*RbcL*	TGGCATCCAAGTTGAAAGAG	ACGCATAAATGGTTGGGAGT
*RbcS*	CCAGGATACTACGATGGGAGG	CTTGTGGGCGATGAAACTGA
*IAA9*	TTCTCTGATGCTATGGATGGATTC	GATTTGGACCGTTCTCATTTGC
*TCP4*	GTCCTCTGCTTCCTCTGATTCG	GATTTCTTCTTCCCACGGTAACG
*PP2C16*	CTACGGTGGCAGTGAATAGTG	CTTCCATCTCTGACCTCTTTCC
*PP2C24*	TCTGATACTGGCGAGCGATG	CCACCACAACCACACTTACG
*PP2C51*	AGAAGCCTGATAGAGAAGATGAAC	ATCCTCGTCACTCCTTGTCG
*SnRK2.8*	TTCGGCTACTCAAAGTCATCAG	CACCAACCAACATCACATATAAGG

*The abbreviations and corresponding full names are as follows: *Tubulin*, Tubulin alpha-3 chain; *RbcL*, Ribulose-1,5-bisphosphate carboxylase/oxygenase large subunit; *RbcS*, Ribulose-1,5-bisphosphate carboxylase/oxygenase large subunit; *IAA9*, Auxin/indole-3-acetic acid 9; *TCP4*, TCP family transcription factor 4; *PP2C16*, Protein Phosphatase 2C 16; *PP2C24*, Protein Phosphatase 2C 24; *PP2C51*, Protein Phosphatase 2C 51; *SNRK2.8*, Sucrose Non-Fermenting 1(SNF1)-Related Protein Kinase 2.8.

### 2.8 Statistical analysis

To investigate the relationships among physiological and biochemical traits under drought stress, we performed correlation analysis, principal component analysis (PCA), hierarchical clustering, and heatmap visualization. Pearson correlation coefficients were calculated based on trait values, and the correlation matrix was visualized using the corrplot package in R. Heatmaps of treatment means (standardized as Z-scores) were generated with the pheatmap package to illustrate clustering patterns of treatments and traits. PCA was conducted to examine the contribution of traits to the principal components. In addition, traits were clustered using the unweighted pair-group method with arithmetic mean (UPGMA), based on a distance matrix derived from Pearson’s correlation (1 – r), to identify groups of traits with similar response patterns.

Statistical analyses were conducted using IBM SPSS Statistics 27 (IBM Corporation, Armonk, NY, USA). For physiological and biochemical indices, including SPAD value, photosynthetic rate, antioxidant enzyme activities (SOD, POD,CAT), MDA content, Proline, soluble protein, soluble sugars, and hormone levels (ABA, IAA), means and standard deviations were calculated for descriptive statistics. All data were first subjected to two-way analysis of variance (ANOVA), followed by Duncan’s new multiple range test at a significance level of *P <* 0.05. In each column, means sharing the same letter (a, b, c, d) are not significantly different (*P* ≥0.05), whereas means with different letters differ significantly (*P <* 0.05).

## 3 Results

### 3.1 The role of ICE6 in regulating photosynthesis in *C. oleifera* seedlings

**Figure 2:**
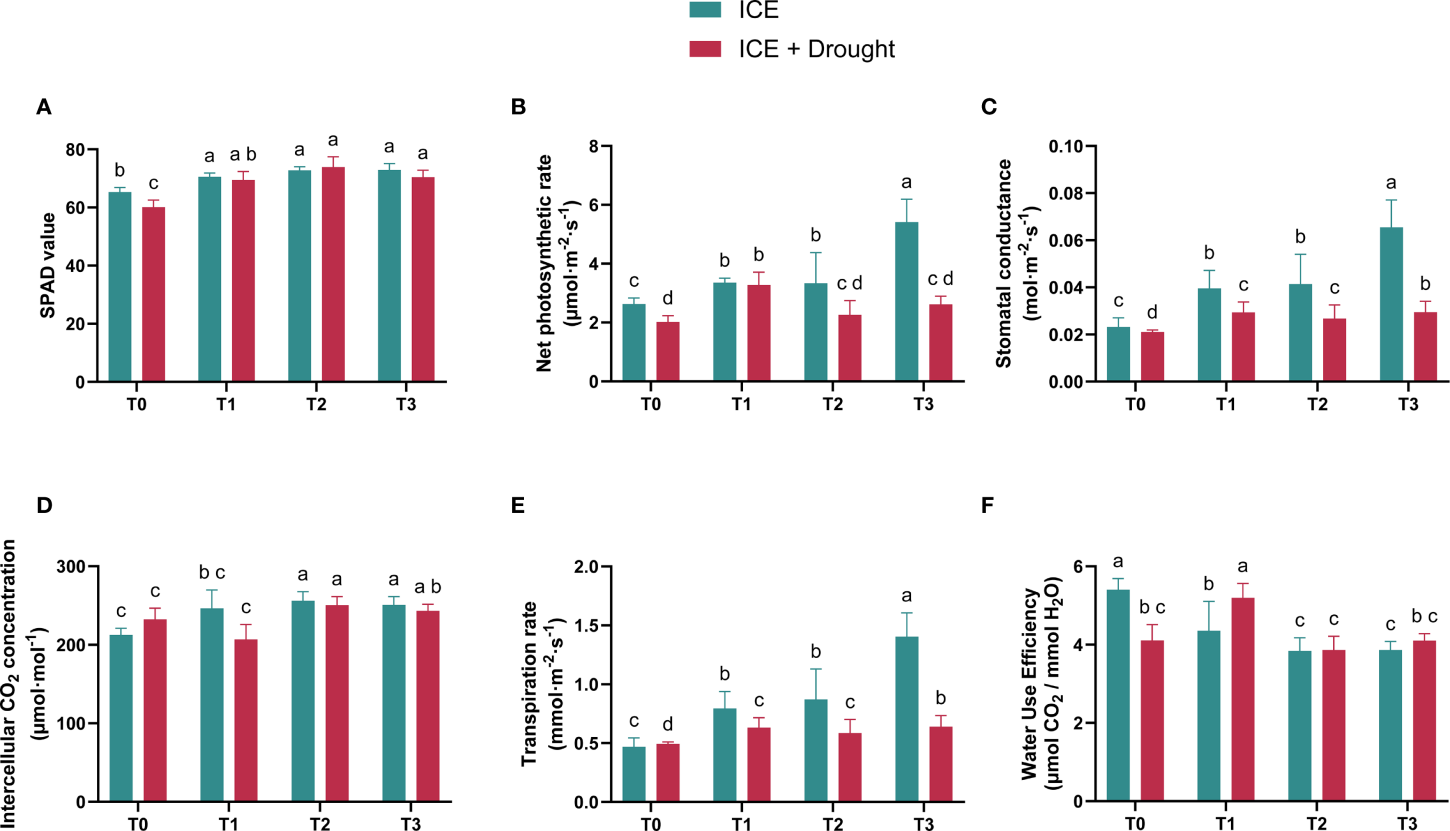
The role of exogenous ICE6 application on the SPAD **(A)**, photosynthetic rate (Pn, **B**), stomatal conductance (Gs, **C**), intercellular CO_2_ concentration (Ci, **D**), transpiration rate (Tr, **E**), and water use efficiency (WUE, **F**) of *C. oleifera* plants. Data are presented as mean ± SD (*n* = 15). Different letters indicate statistical differences (*p <* 0.05) as determined by Duncan’s multiple-range tests for two-way ANOVA. T0: 0 µg/L, T1: 20 µg/L, T2: 40 µg/L, T3: 80 µg/L Iron Chlorin E6 (ICE6).

As illustrated in **Figure 2A**, the SPAD values of *C. oleifera* seedlings significantly decreased under drought conditions compared to normal conditions (T0). Furthermore, the SPAD values of ICE6-treated Camellia oleifera seedlings (T1, T2, T3) were higher than those of the control group (T0) under both normal and drought conditions. Under normal conditions, the SPAD values of ICE6-treated seedlings increased significantly compared to the control, with rises of 7.99% for T1 (20 µg/L), 11.36% for T2 (40 µg/L), and 11.57% for T3 (80 µg/L), corresponding to increasing ICE6 concentrations. Under drought stress, compared to T0, the SPAD values in the ICE6-treated groups remained relatively high, with T2 showing the most notable increase.

Under normal conditions, the Pn of the ICE-treated groups was significantly higher than that of the control group (T0), and Pn increased with higher ICE treatment concentrations. The highest rate was observed in the T3 group, showing a 113.73% increase compared to T0. The patterns of Gs (**Figure 2C**) and Tr (**Figure 2E**) under normal condition were consistent with the Pn. Moreover, the Ci in ICE-treated groups was higher than in T0, with similar Ci values across the different ICE concentrations. Interestingly, WUE was significantly lower in the ICE-treated groups compared to the control group T0 (**Figure 2F**). These observations suggest that treating *C. oleifera* saplings with ICE can significantly enhance their photosynthetic rates.

Under drought stress, the Pn in the T1 and T3 groups was significantly higher than that of the control group (T0), while T2 showed no significant difference compared to the control (**Figure 2B**). Gs and Tr in ICE-treated groups were also significantly higher than in T0, with the lowest Gs and Tr values observed in T2. (**Figures 2C**, **E**). The Ci among ICE-treated groups showed complex variations: Ci in T2 was significantly higher than in T0, while T1 had significantly lower values compared to T0, and T3 exhibited no significant difference from the control (**Figure 2D**). Furthermore, WUE in T1 was significantly higher than in T0, whereas T2 and T3 showed no significant differences from the control (**Figure 2F**). These results suggest that different ICE concentrations elicit varied photosynthetic responses under drought stress. Specifically, T1 was able to sustain higher photosynthetic activity during drought, whereas T2 and T3 appeared to actively downregulate its photosynthetic activity.

### 3.2 The role of ICE6 in regulating hormone responses in *C. oleifera* seedlings

**Figure 3:**
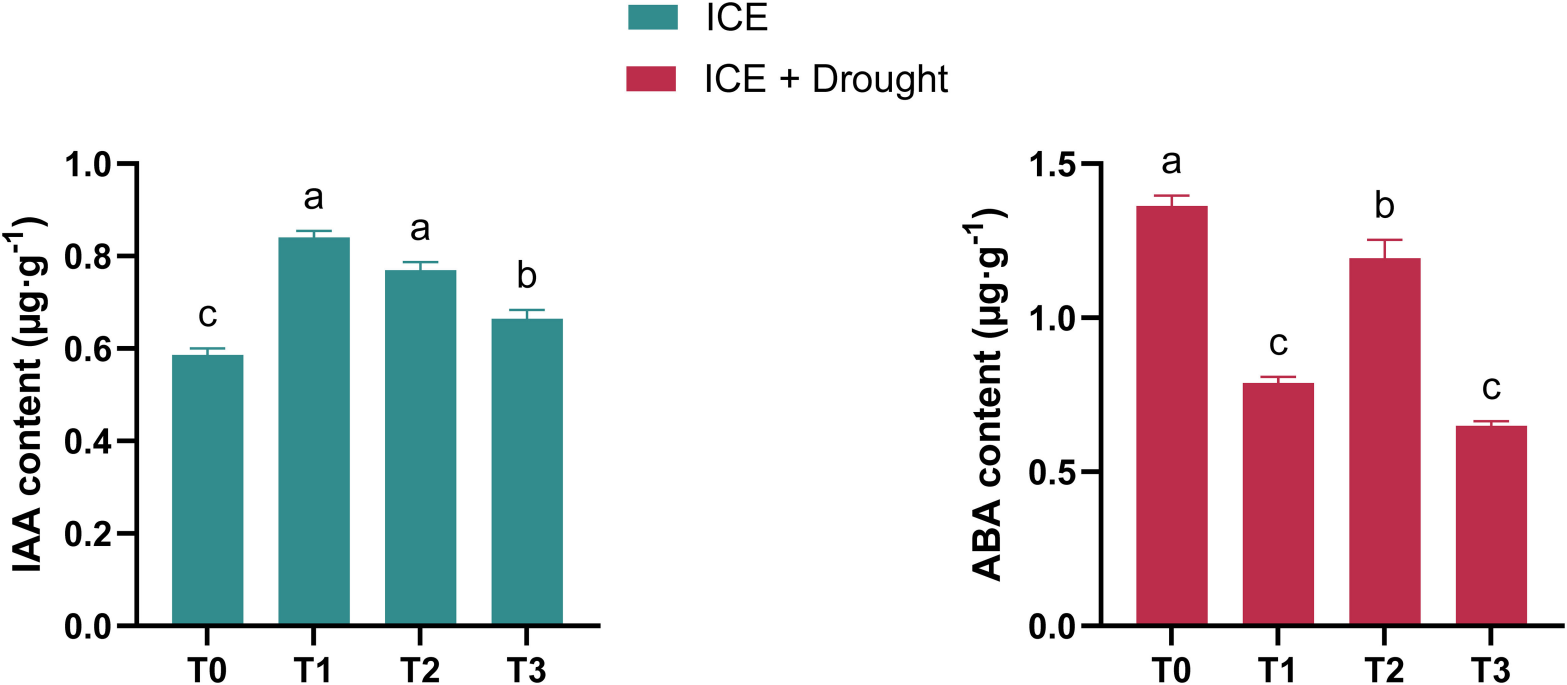
The role of exogenous ICE6 application in regulating hormone responses. Data are presented as mean ± SD (*n* = 3). Different letters indicate statistical differences (*p <* 0.05) as determined by Duncan’s multiple-range tests for two-way ANOVA. T0: 0 µg/L, T1: 20 µg/L, T2: 40 µg/L, T3: 80 µg/L Iron Chlorin E6 (ICE6).

Under normal conditions, the IAA content in the ICE-treated groups was higher than that of T0. However, it gradually decreased as the ICE concentration increased (**Figure 3**). Under drought stress, ABA content in ICE-treated groups was significantly lower than in T0, with T2 exhibiting the highest ABA levels among all treatments, followed by T1 and T3 (**Figure 3**).

### 3.3 The role of ICE6 in regulating the antioxidant system in *C. oleifera* seedlings

Antioxidant enzymes such as SOD, POD, and CAT play crucial roles in maintaining redox balance. They effectively eliminate excess reactive oxygen species (ROS), such as superoxide anions and hydrogen peroxide, which are frequently induced by drought stress (Laxa et al., 2019). These ROS attack unsaturated fatty acids in the cell membranes, triggering lipid peroxidation processes, with malondialdehyde (MDA) being a direct byproduct (Das and Roychoudhury, 2014; Huang et al., 2024). Consequently, an increase in MDA levels typically reflects the level of oxidative stress induced by ROS within the cells (Hasanuzzaman et al., 2020).

**Figure 4:**
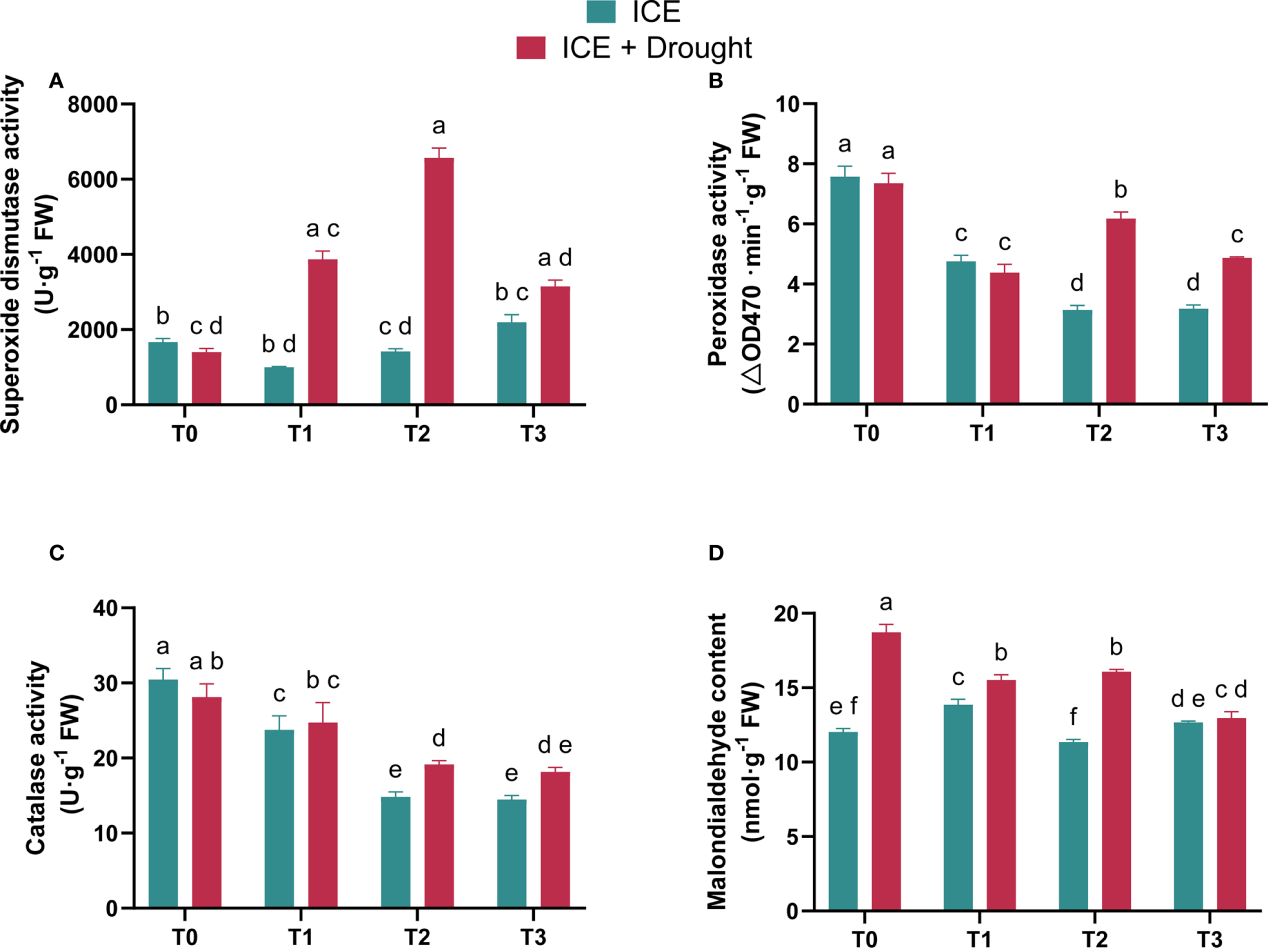
The role of exogenous ICE6 application on superoxide dismutase (SOD, **A**), peroxidase (POD, **B**), catalase (CAT, **C**), and malondialdehyde (MDA, **D**) levels in *C. oleifera* plants Under drought stress. Data are presented as mean ± SD (*n* = 3). Different letters indicate statistical differences (*p <* 0.05) as determined by Duncan’s multiple-range tests for two-way ANOVA. T0: 0 µg/L, T1: 20 µg/L, T2: 40 µg/L, T3: 80 µg/L Iron Chlorin E6 (ICE6).

Under drought stress, SOD activity in ICE-treated groups was significantly higher than that in T0, with the highest activity observed in T2, followed by T1 and T3 (**Figure 4A**). Although the POD (**Figure 4B**) and CAT (**Figure 4C**) activities in ICE-treated *C. oleifera* seedlings were lower than those in the control group (T0) under drought stress, these activities were overall elevated compared to their respective levels under normal conditions. Furthermore, MDA levels in all ICE-treated groups were significantly lower than in T0, with reductions of 18.32% for T1 (20 µg/L), 14.14% for T2 (40 µg/L), and 30.84% for T3 (80 µg/L), indicating enhanced drought tolerance in *C. oleifera* saplings treated with ICE (**Figure 4D**). These results suggest that ICE treatment can effectively activate the antioxidant system in *C. oleifera* seedlings under drought stress. In particular, the T3 treatment showed the lowest MDA level under drought conditions, with no significant difference compared to its respective level under normal conditions.

### 3.4 The role of ICE6 in controlling osmotic regulatory substances in *C. oleifera* seedlings

**Figure 5:**
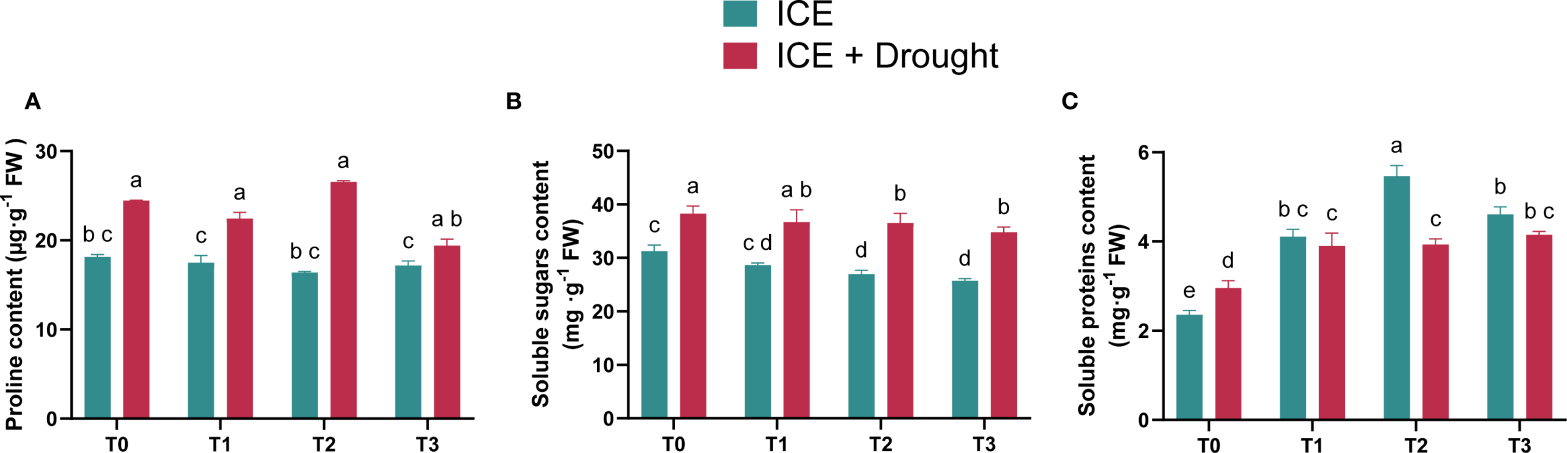
The role of exogenous ICE6 application on proline **(A)**, SS **(B)**, and SP **(C)** contents in *C. oleifera* seedlings. Data are presented as mean ± SD (*n* = 3). Different letters indicate statistical differences (*p <* 0.05) as determined by Duncan’s multiple-range tests for two-way ANOVA. T0: 0 µg/L, T1: 20 µg/L, T2: 40 µg/L, T3: 80 µg/L Iron Chlorin E6 (ICE6).

Under normal conditions, both proline (**Figure 5A**) and soluble sugar levels (**Figure 5B**) in ICE-treated seedlings were not significantly different from those in the control group (T0). However, under drought stress, the proline and soluble sugar levels in C. oleifera seedlings were higher than their respective levels under normal conditions.

Regarding soluble protein (SP) content, under normal conditions, ICE-treated groups exhibited higher SP levels than T0, with the highest content observed in the T2 group, followed by T3 and T1 (**Figure 5C**). Under drought stress, SP content in ICE-treated groups was significantly higher than in T0, indicating that ICE promotes protein accumulation.

### 3.5 RT-qPCR analysis

To investigate how ICE6 influences gene expression in *C. oleifera* saplings, we focused on eight key genes previously identified in our transcriptomic analyses (He et al., 2022). These genes, which show significant differential expression under drought stress, are crucial for understanding hormonal signaling pathways, particularly those involving auxin and ABA.

**Figure 6:**
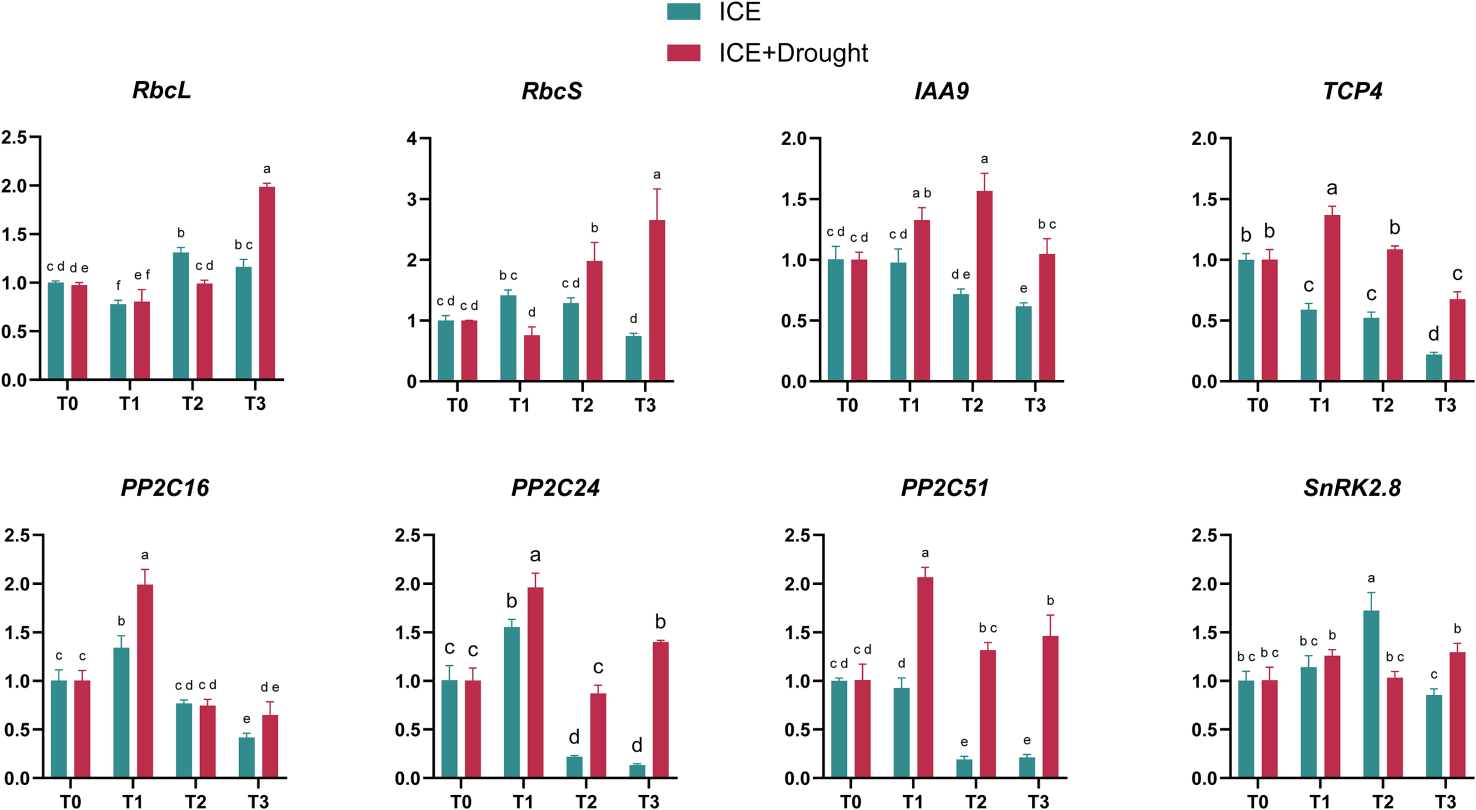
Quantitative Real-Time PCR Analysis of key genes. Different letters indicate statistical differences (*p <* 0.05) as determined by Duncan’s multiple-range tests for two-way ANOVA. The abbreviations and corresponding full names are as follows: *RbcL*, Ribulose-1,5-bisphosphate carboxylase/oxygenase large subunit; *RbcS*, Ribulose-1,5-bisphosphate carboxylase/oxygenase large subunit; *IAA9*, Auxin/indole-3-acetic acid 9; *TCP4*, TCP family transcription factor 4; *PP2C16*, Protein Phosphatase 2C 16; *PP2C24*, Protein Phosphatase 2C 24; *PP2C51*, Protein Phosphatase 2C 51; *SNRK2.8*, Sucrose Non-Fermenting 1(SNF1)-Related Protein Kinase 2.8.

Under normal conditions, the expression levels of *RbcL* in T2 and T3 were significantly higher than the control, while T1 showed slightly lower levels. Meanwhile, *RbcS* expression in T1 and T2 were significantly higher than in the control, whereas T3 was significantly lower (**Figure 6**). Under drought stress, *RbcL* expression in T1 and T2 did not significantly differ from the control, while T3 was significantly higher. *RbcS* expression in T2 and T3 were significantly higher than in the control, with no significant difference observed in T1.

Under normal conditions, *IAA9*, a negative regulator of auxin, exhibited significantly lower expression levels in T2 and T3 compared to the control, while T1 showed no significant difference. However, under drought stress, the highest expression levels of *IAA9* were observed in T2, followed by T1, with T3 showing no significant difference from the control. These results suggest that, under normal conditions, ICE6 treatment in T2 and T3 may activate downstream genes in auxin pathways by inhibiting the negative regulator *IAA9*. Conversely, under drought stress, ICE6 treatment appears to upregulate *IAA9* expression in T1 and T2, which could lead to the suppression of auxin pathway activities. Additionally, expression levels of *TCP4* were significantly reduced across all ICE6-treated groups under normal conditions. During drought stress, *TCP4* expression in T1 was significantly higher than in the control, while T2 showed no significant difference, and T3 was significantly lower. The varied expression of *TCP4* suggests that drought tolerance mechanisms differ across the different ICE6 concentrations.

Under drought stress, the expression levels of *PP2C16*, *PP2C24*, *PP2C51*, and *SnRK2.8* in T1 were higher than in the control, indicating enhanced ABA signaling. In T3, expression levels of *PP2C24*, *PP2C51*, and *SnRK2.8* were also significantly higher than in the control. However, in T2, the expression of these genes showed no significant differences compared to the control.

### 3.6 Principal component analysis and trait correlations

**Figure 7:**
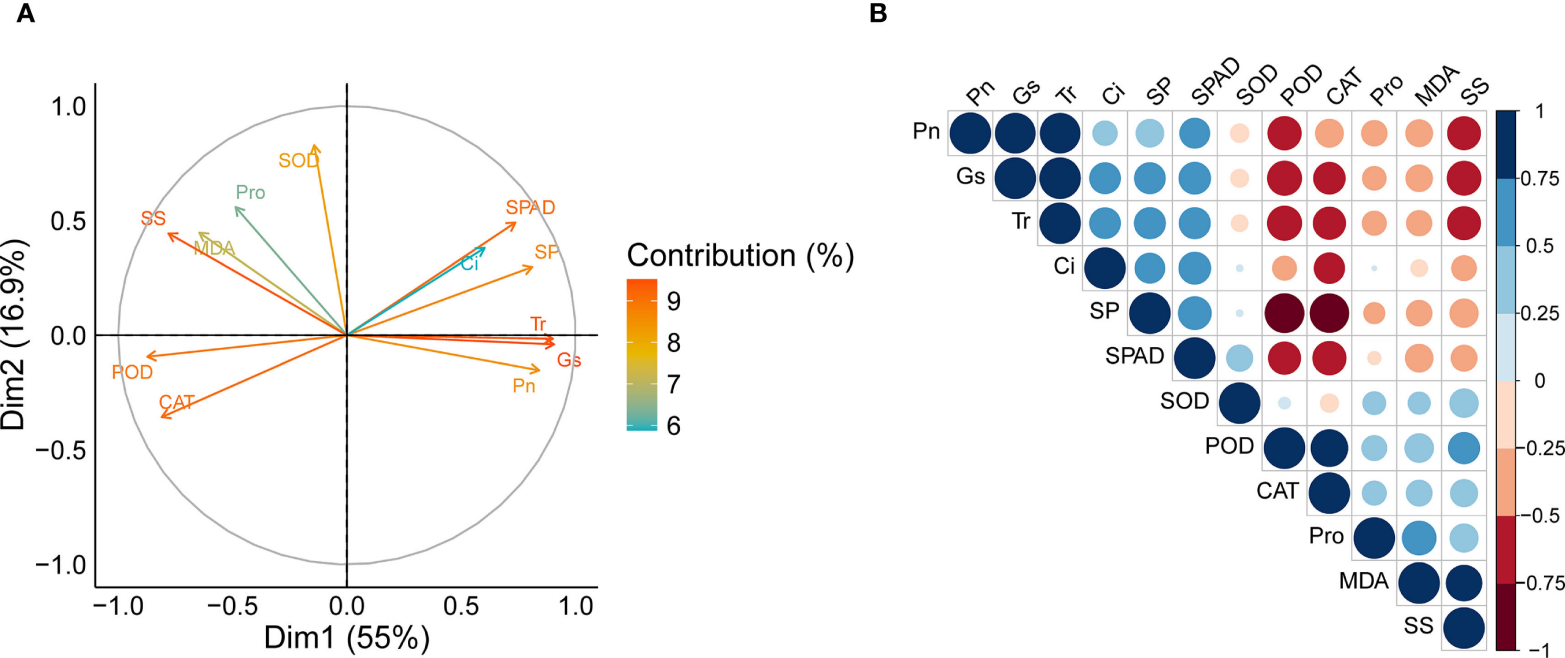
PCA variable plot and correlations among traits. **(A)** PCA variable plot showing the contributions of each phenotypic trait to the principal components. **(B)** Pearson correlation matrix among phenotypic traits. SOD, superoxide dismutase activity; POD, peroxidase activity; CAT, catalase activity; MDA, malondialdehyde content; Pro, proline content; SS, soluble sugar content; SP, soluble protein content; SPAD, chlorophyll content index; Pn, net photosynthetic rate; Ci, intercellular CO_2_ concentration; Gs, stomatal conductance; Tr, transpiration rate.

Principal component analysis (PCA) revealed that the first two principal components (PC1 and PC2) explained 54.98% and 16.95% of the total variance, respectively, accounting for a cumulative variance of 71.93% (**Figure 7A**). The enzymatic antioxidant traits such as POD and CAT were strongly associated with the negative axis of PC1, whereas SOD was mainly associated with PC2. Lipid peroxidation marker MDA, osmolyte Pro and SS showed moderate contributions to both PC1 and PC2. Photosynthetic traits such as SPAD, Pn, Gs, and Tr were positioned opposite to POD and CAT vectors, indicating negative associations with these enzymatic antioxidant traits along PC1. This pattern was further supported by the Pearson correlation heatmap (**Figure 7B**), in which POD and CAT were positively correlated with each other but negatively correlated with SPAD, Pn, Gs, and Tr. Photosynthetic traits showed strong positive intercorrelations, while MDA and Pro exhibited moderate positive correlations with POD and CAT and negative correlations with most photosynthetic parameter.

### 3.7 Cluster analysis of physiological and biochemical traits

**Figure 8:**
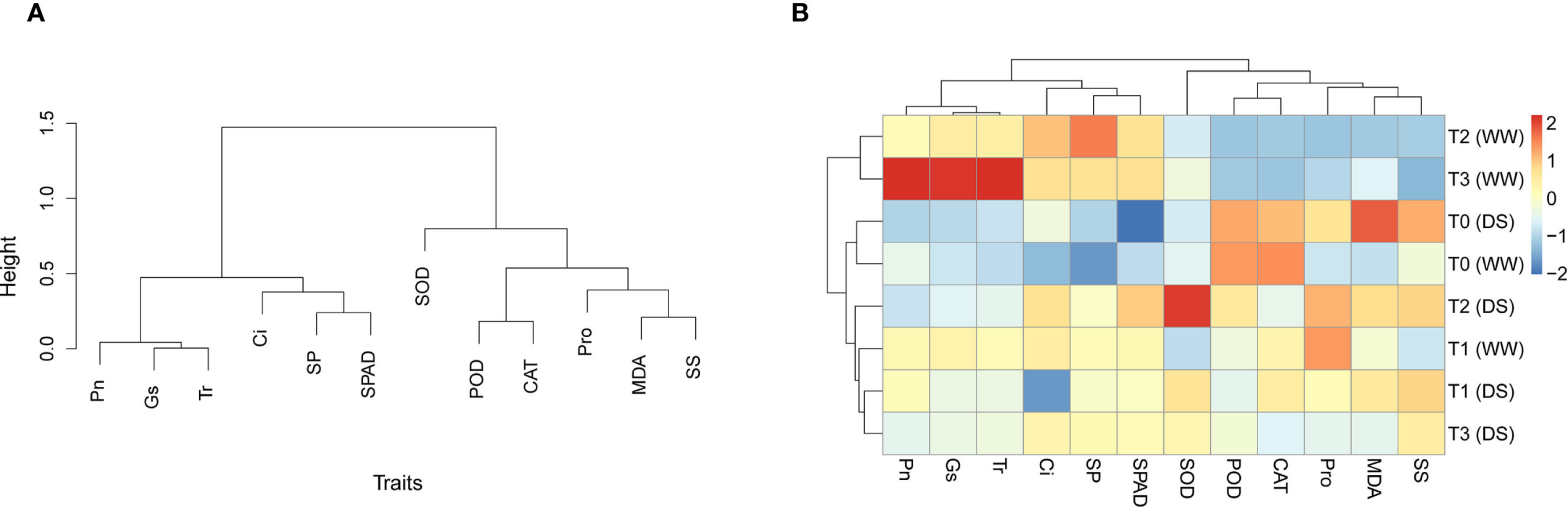
The UPGMA dendrogram **(A)** and the heatmap **(B)** of physiological and biochemical traits under drought stress. SOD, superoxide dismutase activity; POD, peroxidase activity; CAT, catalase activity; MDA, malondialdehyde content; Pro, proline content; SS, soluble sugar content; SP, soluble protein content; SPAD, chlorophyll content index; Pn, net photosynthetic rate; Ci, intercellular CO_2_ concentration; Gs, stomatal conductance; Tr, transpiration rate; WW, well-watered; DS, drought stress; T1, 20 µg/L; T2, 40 µg/L; T3, 80 µg/L.

The UPGMA dendrogram grouped the physiological and biochemical traits into two major clusters (**Figure 8A**). Photosynthetic-related parameters (SPAD, Pn, Gs, Tr) were closely associated, while oxidative stress and antioxidant indicators (MDA, SOD, POD, CAT, Pro, etc.) formed another cluster. This classification highlights the coordinated responses of photosynthetic traits and stress-related traits under drought conditions. Consistently, the heatmap of standardized traits revealed distinct patterns among treatments (**Figure 8B**). Under well-watered conditions, T3 exhibited the most favorable profile, with the highest SPAD, Pn, Gs, and Tr, indicating optimal photosynthetic performance under non-stress conditions. Under drought stress, T3 also showed the lowest MDA while largely maintaining photosynthetic traits, suggesting effective mitigation of oxidative damage. Overall, T3 demonstrated superior performance under both normal and drought stress conditions.

## 4 Discussion

In this study, exogenous application of ICE6 significantly influenced the photosynthesis and physiological characteristics of *C. oleifera* saplings. The results indicated that, under normal conditions, the photosynthetic rate, SPAD value, and soluble protein content in ICE6-treated *C. oleifera* plants were significantly higher compared to the control (T0). These results may be related to ICE6’s ability to promote chlorophyll synthesis and inhibit its degradation, thereby enhancing the photosynthetic capacity of *C. oleifera saplings* (Xing et al., 2020). In this study, the T3 (80 µg/L) treatment group exhibited performance distinctly different from T1 (20 µg/L) and T2 (40 µg/L) under normal conditions, as demonstrated by its significantly higher net photosynthesis rates compared to T1 (20 µg/L) and T2 (40 µg/L) (**Figure 2B**). This suggests that T3 (80 µg/L) may enhance photosynthesis through mechanisms other than stomatal opening, even though there was no significant change in the Ci. This enhancement is likely related to the optimization of light capture and conversion in T3 (80 µg/L), such as increased activity of the Rubisco enzyme, improvements in chloroplast structure, and enhanced functionality of the photosynthetic electron transport chain, thereby enhancing the efficiency of CO_2_ utilization in photosynthesis (Qiao et al., 2024; Croce et al., 2024). In addition, the WUE of ICE6-treated groups was significantly lower than that of the control, suggesting that ICE6-treated saplings may prioritize enhancing photosynthesis over maintaining WUE under normal conditions. Furthermore, the IAA content in ICE6-treated saplings was higher, while soluble sugar content was lower compared to the control, which may have directly facilitated seedling growth. As *IAA9* is a known negative regulator of auxin, its reduced expression leads to increased auxin activity, promoting cell elongation and division (Wang et al., 2005). In this study, the expression of *IAA9* (**Figure 6**) were generally lower in ICE6-treated saplings than in the control under normal conditions, particularly in T2 (40 µg/L) and T3 (80 µg/L), further confirming enhanced auxin activity.

Previous studies in olive and pear tree have indicated that under drought stress, a reduction in RuBisCO activity and chlorophyll content often leads to a significant decrease in photosynthetic rate (Hao et al., 2023; Boussadia et al., 2023). In this study, ICE6-treated *C. oleifera* plants maintained relatively high SPAD values and photosynthetic activity under drought conditions, suggesting that ICE6 can mitigate the adverse effects of drought to some extent. Notably, the T2 (40 µg/L) group had the highest SPAD values, and *RbcS* expression levels were also higher than in the control under drought stress. However, the photosynthetic rate showed no significant difference from the control. This implies that the T2 (40 µg/L) group may have actively reduced its photosynthetic rate to prioritize survival over growth. Furthermore, *IAA9* expression levels in T2 (40 µg/L) were significantly higher than those in the control, suggesting that T2 (40 µg/L) plants adopted a strategy of limiting growth activity to ensure survival under drought conditions. This may also be linked to the elevated ABA content observed in the T2 (40 µg/L) group. As a key hormone involved in drought stress response, ABA mediates stomatal closure, which may further decrease the photosynthetic rate (Lim et al., 2015; Li et al., 2022).

Moreover, although ABA content in T1 and T3 groups was significantly lower than in the control group under drought stress, expression levels of *PP2C24*, *PP2C51*, and *SnRK2.8* were significantly higher. *PP2C* protein phosphatases and *SnRK2.8* are key components of the ABA signaling pathway (Nakashima and Yamaguchi-Shinozaki, 2013; Ma et al., 2009; Soon et al., 2012). The high expression of these genes suggests that *C. oleifera* saplings in T1 (20 µg/L) and T3 (80 µg/L) may still utilize the ABA signaling pathway to regulate stress responses and enhance drought tolerance. In contrast, while T2 (40 µg/L) had higher ABA content, expression levels of *PP2C24*, *PP2C51*, and *SnRK2.8* were not significantly different from the control, indicating that T2 (40 µg/L) might not primarily rely on the ABA signaling pathway for its drought response. Instead, T2 (40 µg/L) showed higher SOD and POD enzyme activities that are crucial for scavenging excess ROS produced under drought stress. In this study, due to its higher antioxidant enzyme activities, the MDA content in T2 (40 µg/L) was significantly lower than that in the control group, indicating its effectiveness in mitigating lipid peroxidation and protecting cells from oxidative stress caused by drought.

Previous studies have suggested that under drought stress, plants typically increase proline and soluble sugar content to regulate osmotic pressure, mitigate water loss, and reduce oxidative damage (Dien et al., 2019; Ozturk et al., 2021). In this study, although there were no significant differences in soluble sugar content among the groups under drought conditions, the soluble sugar content increased compared to normal growth conditions (**Figure 5B**). Similarly, the proline content in the drought-treated groups was higher than under normal growth conditions (**Figure 5A**). The changes in soluble sugar and proline content observed in this study are generally consistent with classical drought response findings (Khedr et al., 2003). Interestingly, the T2 group exhibited the lowest proline content among all treatments under normal conditions. However, under drought stress, the proline content in the T2 (40 µg/L) was highest. This suggests that the T2 (40 µg/L) group may rely more on proline to enhance osmotic adjustment, thereby improving its adaptation to drought conditions (Furlan et al., 2020).

Furthermore, under drought stress, the expression levels of *TCP4* provided insights into the differential drought tolerance of plants treated with varying ICE6 concentrations. *TCP4* is a positive regulator of plant response to water stress, and higher *TCP4* expression is known to enhance plant tolerance (Wang et al., 2022). Therefore, the elevated TCP4 levels in T1 (20 µg/L) suggest stronger drought tolerance in this group (**Figure 6**). In contrast, although *TCP4* expression in T3 (80 µg/L) was significantly lower than in the control, the photosynthetic parameters and key physiological indicators such as MDA remained relatively stable conditions. This stability may be attributed to the enhanced drought adaptability in T3 (80 µg/L) mediated through the ABA signaling pathway.

In previous studies, ICE6 has been mainly applied as a plant growth regulator under non-stress conditions, where it promoted the growth of crops such as ginger (Shengqi et al., 2024), tobacco (Xing et al., 2020), and rice (Xie et al., 2022). In this study, we demonstrate that ICE6 also enhances drought tolerance in *C. oleifera*, as evidenced by enhanced antioxidant enzyme activities, reduced MDA levels, and the maintenance of relatively stable photosynthetic performance. This is consistent with previous ICE6 studies on soybean (Cao CunFeng et al., 2018) and rapeseed plants (Cao et al., 2016) under salt stress conditions. This opens up promising avenues for utilizing ICE6 to improve agricultural productivity and sustainability, potentially reducing the need for water and chemical inputs in stressed environments. However, due to the limitations of pot experiments, these findings need further validation through extensive long-term field trials. Additionally, it is crucial to further explore the molecular mechanisms of ICE6, particularly its interactions with other plant hormones and its efficacy under multiple stress conditions. Such studies will help fully elucidate the potential of ICE6 and provide a scientific basis for the breeding of *C. oleifera* varieties with enhanced stress resistance.

## 5 Conclusions

This study preliminarily explored the influence of exogenous ICE6 on the physiological and biochemical responses of *C. oleifera* seedlings under normal and drought conditions. The results indicate that ICE6 significantly enhances the photosynthetic rate, SPAD value, and soluble protein content of the seedlings, thereby potentially promoting their growth. Additionally, ICE6 treatment enhanced the drought tolerance of *C. oleifera* seedlings, as evidenced by higher antioxidant enzyme activities, lower malondialdehyde (MDA) content, and stable photosynthetic parameters. These responses are likely related to mechanisms such as promoting chlorophyll synthesis, regulating the ABA signaling pathway, and increasing proline and protein accumulation. Overall, ICE6 at 80 µg/L was optimal, increasing net photosynthetic rate by 113.7% and SPAD by 11.6% under normal irrigation, while reducing MDA by 30.8% during drought stress, demonstrating its efficacy as a plant growth regulator for enhancing both growth and stress tolerance.

## Data Availability

The raw data supporting the conclusions of this article will be made available by the authors, without undue reservation.
