# Supplementary material for: Iron Chlorin E6 enhances drought resilience in Camellia oleifera Abel
Source: Front Plant Sci. 2025 Sep 15;16:1666016. doi: 10.3389/fpls.2025.1666016 (PMC12477230; doi:10.3389/fpls.2025.1666016)
Supplement: Supplementary file 2 [file DataSheet1.docx]

1. **ABA**

**ABA Standard Curve:**

| **Index Name** | **Standard Curve** | **Retention Time（min）** |
| --- | --- | --- |
| ABA | y = 33342x - 750.8, R2 = 0.9968 | 33.412 |
| Y：Peak Area, x: Concentration | | |


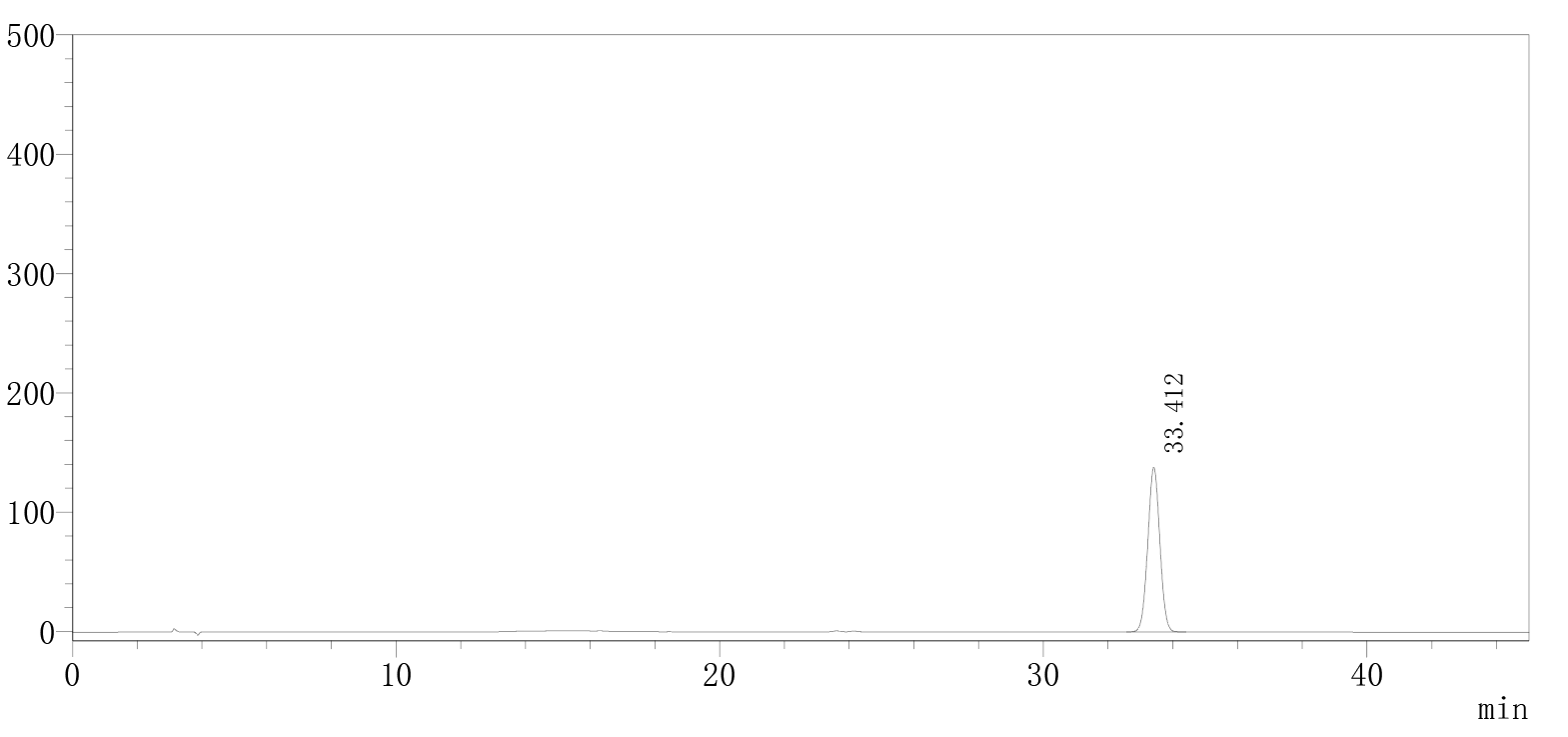


**Figure 1.** Chromatogram of ABA Standard

**Table 1.** ABA Quantification Results for Different Samples

| **Sample** | **Mass (g)** | **Peak Area** | **ABA content (μg/g)** |
| --- | --- | --- | --- |
| T0-1 | 0.2051 | 28787 | 1.296 |
| T0-2 | 0.2050 | 31311 | 1.407 |
| T0-3 | 0.2044 | 30726 | 1.386 |
| T1-1 | 0.2051 | 16278 | 0.747 |
| T1-2 | 0.2045 | 17456 | 0.801 |
| T1-3 | 0.2047 | 17792 | 0.815 |
| T2-1 | 0.2004 | 27984 | 1.290 |
| T2-2 | 0.2011 | 26192 | 1.205 |
| T2-3 | 0.2016 | 23509 | 1.083 |
| T3-1 | 0.2033 | 13916 | 0.649 |
| T3-2 | 0.2031 | 13299 | 0.622 |
| T3-3 | 0.2035 | 14520 | 0.675 |

1. **IAA**

**IAA Standard Curve:**

| **Index Name** | **Standard Curve** | **Retention Time（min）** |
| --- | --- | --- |
| IAA | y = 10182x - 33.3, R2 = 0.9967 | 20.646 |
| Y：Peak Area, x: Concentration | | |


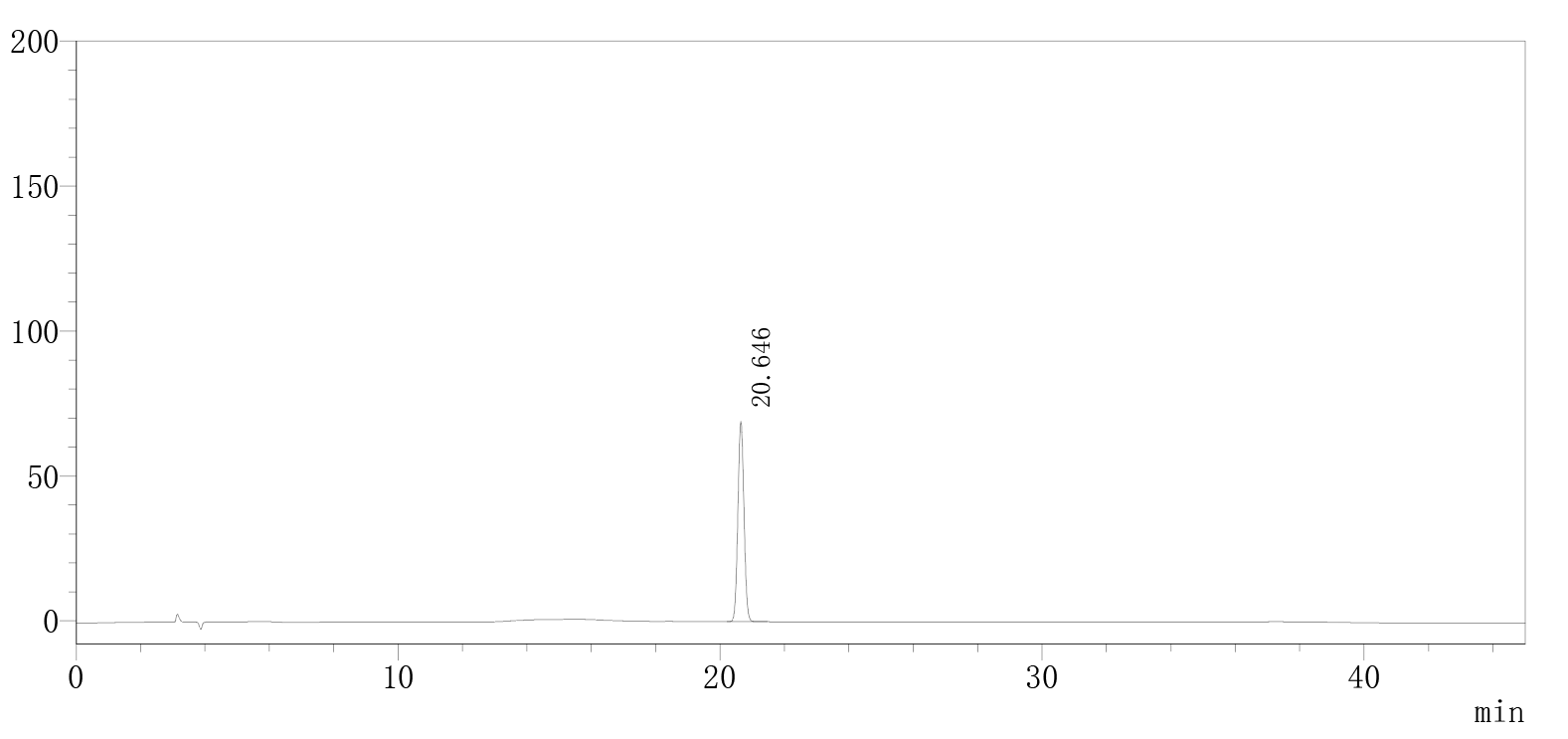


**Figure 2.** Chromatogram of IAA Standard

**Table 2.** IAA Quantification Results for Different Samples

| **Sample** | **Mass (g)** | **Peak Area** | **IAA content (μg/g)** |
| --- | --- | --- | --- |
| T0-1 | 0.2033 | 3988 | 0.583 |
| T0-2 | 0.2031 | 3852 | 0.564 |
| T0-3 | 0.2035 | 4196 | 0.612 |
| T1-1 | 0.2008 | 5732 | 0.846 |
| T1-2 | 0.2010 | 5511 | 0.813 |
| T1-3 | 0.2012 | 5850 | 0.862 |
| T2-1 | 0.2066 | 5602 | 0.804 |
| T2-2 | 0.2056 | 5216 | 0.752 |
| T2-3 | 0.2061 | 5238 | 0.754 |
| T3-1 | 0.2078 | 4379 | 0.626 |
| T3-2 | 0.2071 | 4757 | 0.682 |
| T3-3 | 0.2068 | 4782 | 0.686 |
